# Mobile Health App With Social Media to Support Self-Management for Patients With Chronic Kidney Disease: Prospective Randomized Controlled Study

**DOI:** 10.2196/19452

**Published:** 2020-12-15

**Authors:** Wen-Yi Li, Fu-Chun Chiu, Jyun-Kai Zeng, Yao-Wei Li, Su-Hua Huang, Hui-Chin Yeh, Bor-Wen Cheng, Feng-Jung Yang

**Affiliations:** 1 Renal Division Department of Internal Medicine National Taiwan University Hospital Yun Lin Branch Douliu Taiwan; 2 School of Medicine, College of Medicine National Taiwan University Taipei Taiwan; 3 Cardiovascular Division Department of Internal Medicine National Taiwan University Hospital Yun Lin Branch Douliu Taiwan; 4 Department of Industrial Engineering and Management National Yunlin University of Science and Technology Douliu Taiwan; 5 Department of Dietetics National Taiwan University Hospital Yun Lin Branch Douliu Taiwan; 6 Department of Applied Foreign Languages National Yunlin University of Science and Technology Douliu Taiwan

**Keywords:** chronic kidney disease, self-management, self-efficacy, quality of life, health management platform, wearable device

## Abstract

**Background:**

Chronic kidney disease (CKD) is a global health burden. Self-management plays a key role in improving modifiable risk factors.

**Objective:**

The aim of this study was to evaluate the effectiveness of wearable devices, a health management platform, and social media at improving the self-management of CKD, with the goal of establishing a new self-management intervention model.

**Methods:**

In a 90-day prospective experimental study, a total of 60 people with CKD at stages 1-4 were enrolled in the intervention group (n=30) and control group (n=30). All participants were provided with wearable devices that collected exercise-related data. All participants maintained dietary diaries using a smartphone app. All dietary and exercise information was then uploaded to a health management platform. Suggestions about diet and exercise were provided to the intervention group only, and a social media group was created to inspire the participants in the intervention group. Participants’ self-efficacy and self-management questionnaire scores, Kidney Disease Quality of Life scores, body composition, and laboratory examinations before and after the intervention were compared between the intervention and control groups.

**Results:**

A total of 49 participants completed the study (25 in the intervention group and 24 in the control group); 74% of the participants were men and the mean age was 51.22 years. There were no differences in measured baseline characteristics between the groups except for educational background. After the intervention, the intervention group showed significantly higher scores for self-efficacy (mean 171.28, SD 22.92 vs mean 142.21, SD 26.36; *P*<.001) and self-management (mean 54.16, SD 6.71 vs mean 47.58, SD 6.42; *P*=.001). Kidney Disease Quality of Life scores were also higher in the intervention group (mean 293.16, SD 34.21 vs mean 276.37, SD 32.21; *P*=.02). The number of steps per day increased in the intervention group (9768.56 in week 1 and 11,389.12 in week 12). The estimated glomerular filtration rate (eGFR) of the intervention group was higher than that of the control group (mean 72.47, SD 24.28 vs mean 59.69, SD 22.25 mL/min/1.73m^2^; *P*=.03) and the decline in eGFR was significantly slower in the intervention group (–0.56 vs –4.58 mL/min/1.73m^2^). There were no differences in body composition between groups postintervention.

**Conclusions:**

The use of wearable devices, a health management platform, and social media support not only strengthened self-efficacy and self-management but also improved quality of life and a slower eGFR decline in people with CKD at stages 1-4. These results outline a new self-management model to promote healthy lifestyle behaviors for patients with CKD.

**Trial Registration:**

ClinicalTrials.gov NCT04617431; https://www.clinicaltrials.gov/ct2/show/NCT04617431

## Introduction

Chronic kidney disease (CKD) is a global public health issue. CKD leads to end-stage renal disease (ESRD), and the dialysis therapy associated with ESRD incurs a huge economic burden for many countries. In Taiwan, the incidence and prevalence of ESRD are among the highest in the world [1]. Taiwan’s national prevalence of CKD is estimated at 11.93% [2], which is largely due to low public awareness of the problem.

CKD is a lifelong health condition, and people with CKD often have other comorbidities such as hypertension, diabetes, and heart disease. Care for CKD involves a multidisciplinary team of researchers, engineers, and clinicians. A Cochrane database systematic review showed evidence for significant beneficial effects of regular exercise on physical fitness, walking capacity, cardiovascular dimensions (eg, blood pressure and heart rate), health-related quality of life, and nutritional parameters in adults with CKD [3]. Another systematic review showed that a combined exercise and dietary intervention resulted in a slower decline of the estimated glomerular filtration rate (eGFR) in patients with diabetes and CKD stages 3-5 [4]. However, people with CKD often feel constrained by the physical discomfort, complex treatment regimes, side effects, and liquid and dietary restrictions associated with the disease [5]. People at any stage of the disease are recommended to maintain healthy regimes of diet and exercise, along with good adherence to the medication. The ability to monitor one’s lifestyle not only promotes health but also reduces the cost of health care.

Self-management is an important factor for helping people cope with chronic diseases [6]. The five fundamental self-management skills include problem-solving, decision-making, resource utilization, formation of client–health care provider partnerships, and taking action [7]. Self-efficacy is a crucial mediator between knowledge and self-care [8], and providing effective self-management support is a key policy that aims to improve the skills and confidence of patients in managing their illness [9].

In 2008, Costantini et al [10] examined the self-management experiences of people with mild to moderate CKD (stages 1-3). They found that participants wanted to self-manage their illness in collaboration with their health care providers; thus, people with early CKD need guidance and support from health care professionals to successfully self-manage their treatment [10]. In 2018, Wu et al [11] demonstrated that an innovative self-management intervention effectively decreased the serum creatinine levels and depression symptoms in people with CKD. Strengthening self-management skills has been shown to lead to a delay in the progression of CKD through improving modifiable risk factors [12].

A health management platform uses information and big data to provide the user with the ability to analyze, detect, monitor, and control risk factors of a disease. Hardinge et al [13] used a home-based mobile health (mHealth) platform for reporting daily symptoms and medication use, and for measuring physiological variables such as pulse rate and oxygen saturation in patients with chronic obstructive pulmonary disease, providing evidence for integrating telehealth interventions with clinical care pathways to support self-management.

The use of apps—software programs that run on mobile devices—is a recent approach for delivering health information and education to patients [14]. Studies have shown that a diabetes-related smartphone app combined with weekly SMS text messaging support from a health care professional could significantly improve glycemic control in adults with type 1 diabetes [15]. In a prior study, we found potential of a primary physician–led telehealth care model based on a social network service in delaying dialysis initiation for patients with stage-5 CKD [16]. According to a survey from app platforms, 67 out of 177 apps were recommended for CKD patients [17]. The most common functionalities used were CKD information and self-management (57%), e-consultation (25%), and CKD nutrition education (24%). However, the continuity of patient-centered care for CKD provided by mHealth apps is currently inadequate [10].

According to 2017 data from the Institute for Information Industry, in Taiwan, 60.2% of people older than 55 years owned a smartphone [18]. LINE is the predominant text messaging app, which is used by approximately 66.6% of people in Taiwan in their daily lives. However, there have been few interventional studies conducted to date involving the use of either wearable devices to quantify motor performance or an app-based platform to interact with CKD patients. Thus, we conducted this study to evaluate the ability of a health management platform with a wearable device and social media platform to improve participants’ self-management abilities and delay the progression of CKD.

## Methods

### Study Design

This study was a two-arm randomized controlled trial with a pretest-posttest design. The study protocol was approved by the Research Ethics Committee of National Taiwan University Hospital (No. 201808094RINB). The trial is registered at ClinicalTrials.gov (NCT04617431). Written informed consent was obtained from all participants before starting the study. All research procedures followed the directives of the Declaration of Helsinki.

### Study Population

CKD is defined based on abnormalities of kidney function or structure for more than 3 months. The different stages of CKD form a continuum as follows [19]: stage 1, kidney damage with normal or increased eGFR (>90 mL/min/1.73 m^2^); stage 2, mild reduction in eGFR (60-89 mL/min/1.73 m^2^); stage 3a, moderate reduction in eGFR (45-59 mL/min/1.73 m^2^); stage 3b, moderate reduction in eGFR (30-44 mL/min/1.73 m^2^); stage 4, severe reduction in eGFR (15-29 mL/min/1.73 m^2^); and stage 5, kidney failure (eGFR <15 mL/min/1.73 m^2^ or dialysis).

This study prospectively enrolled patients with CKD stages 1-4 from the nephrology outpatient clinic of National Taiwan University Hospital Yunlin branch between January 2019 and May 2019, which is a regional teaching hospital located in a suburban area in central Taiwan. The patients were cared for by their primary care nephrologists according to the Kidney Disease Outcomes Quality Initiative guidelines [20]. Inclusion criteria were aged ≥20 years and a diagnosis of CKD stage 1-4. Those who agreed to participate in the study signed informed consent forms. Exclusion criteria were an inability to use a smartphone, impaired walking capacity, a psychiatric disorder, or any hospitalization during the previous 3 months. Participants were assigned randomly to the intervention group or the control group. We performed stratified sampling according to CKD stage and enrolled more participants at stages 2 and 3 than at stage 4. The nephrologists were blind to group allocation, whereas the investigators and participants were not.

### Sample Size

G-Power 3.1.9.4 was used to calculate the sample size on the basis of an effect size of 0.35 according to the related literature [21]. To obtain a power of 0.80, α of .05, and effect size of 0.35, according to a two-tailed test, the required sample size was determined to be at least 44 (with 22 participants in each group). We estimated a 20% attrition rate, and therefore the total sample required at least 53 participants (27 in each group).

### Data Collection

A brief interview was conducted with all participants, assisted by an electronic medical record search, to document their demographic profiles and comorbidities. The diagnosis of any comorbidity was documented by clinically relevant history, medical examinations, or pathological reports. Body composition was assessed using a body composition analyzer (Omron HBF-701), including body height, body weight, and percentage body fat measurements. Participants completed self-efficacy and self-management questionnaires and the Kidney Disease Quality of Life survey (KDQOL-SF), which includes the 36-item Short Form Health Survey. Laboratory data, including hemogram, serum biochemistry, electrolyte profile, and renal function assay, were measured as per routine care for CKD patients according to the guidelines of the Taiwan Society of Nephrology.

### Outcome Measurement Instruments

The self-efficacy questionnaire [22] is composed of eight subscales: blood sugar or blood pressure control, diet, exercise, medication, lifestyle, infection prevention, problem-solving, and partnership. The scale has 20 questions; scores range from 0 to 10 and reflect the level of confidence, where 0 is “completely without confidence” and 10 is “completely confident.” The total scores ranged between 0 and 200, and a higher score indicates that the patient has greater confidence in controlling their disease. The Cronbach α was .81.

The self-management questionnaire [22] is composed of four subscales: partnership, compliance, self-care, and problem-solving. There are 16 questions with 4-point Likert items (1, “never”; 2, “sometimes”; 3, “usually”; and 4, “always”). The total scores ranged between 16 and 64, and a higher score indicates better self-management ability. The Cronbach α was .81.

Quality of life was measured by the KDQOL-SF [23] (dialysis version). This instrument was developed for patients with kidney disease who are on dialysis and has been adapted for nondialysis patients [24]. The KDQOL-SF includes the 36-item Short Form Health Survey, supplemented with multi-item scales targeted at particular concerns of individuals with kidney disease (eg, symptoms/problems, effects of kidney disease on daily life, burden of kidney disease, cognitive function, work status, sexual function, quality of social interaction, and sleep). The total scores ranged between 74 and 360, with a higher score indicates a better quality of life. The Cronbach α was between .61 and .90.

### Intervention

**Figure 1:**
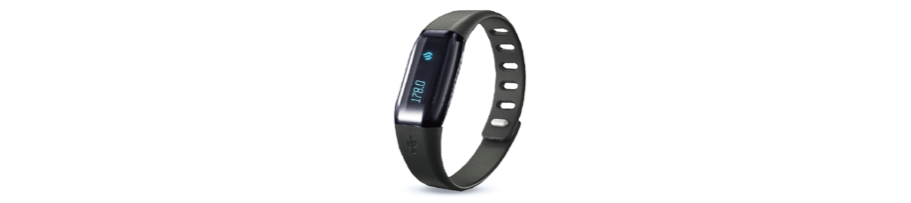
Heart Rate Smart Wristband, GSH405-B6, Golden Smart Home Technology Corporation.

Each participant was provided with a wearable device (Heart Rate Smart Wristband, GSH405-B6, Golden Smart Home Technology Corporation) (Figure 1). The wristband was approved by the National Communications Commission of Taiwan (NCC verification code: CCAB16LP1430T3). This wristband can detect steps (0-120,000 steps, division 1 step), calories, and sleep, and was validated in our previous study [25].

**Figure 2:**
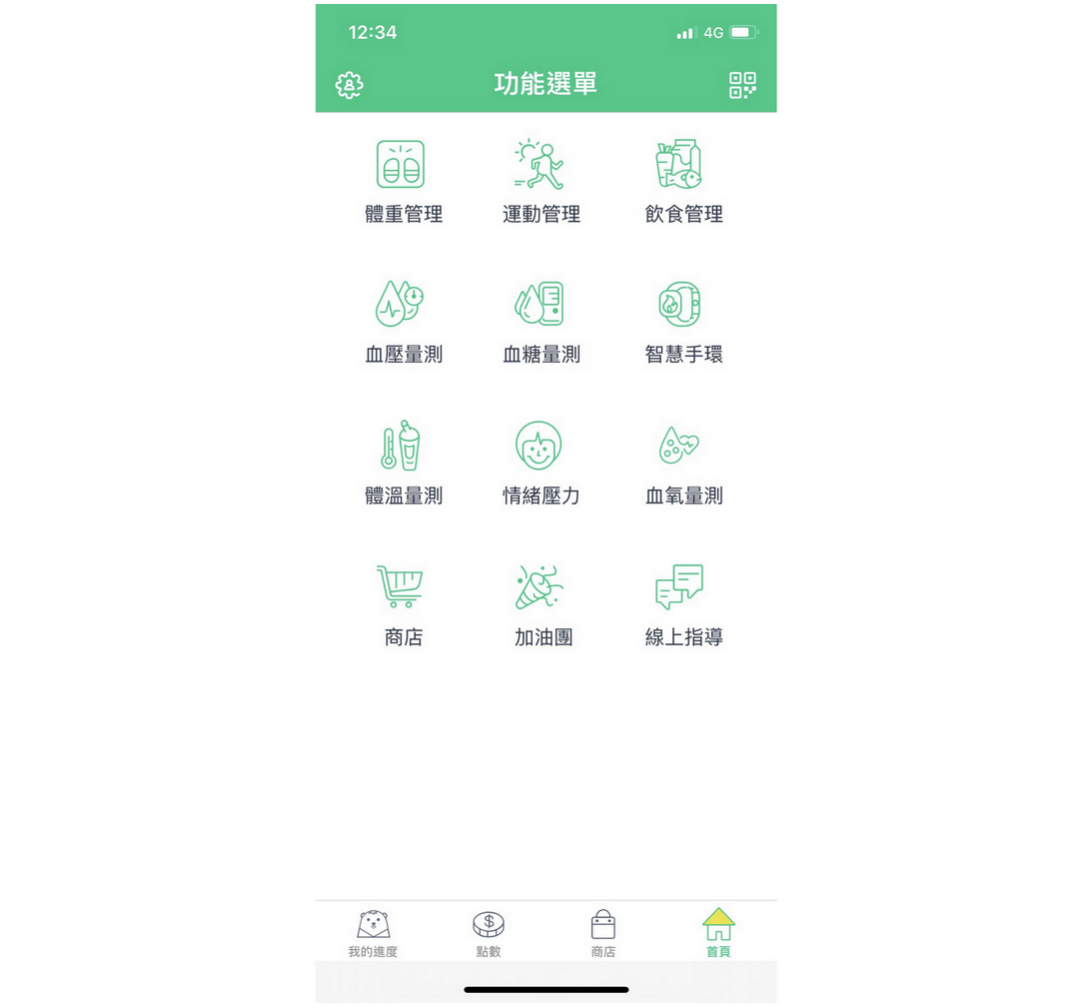
User interface of the health management platform software (WowGoHealth app).

Each participant downloaded the WowGoHealth app (Figure 2) to connect with the health management platform (GSH AI health platform). Participants’ exercise-related data, including the number of steps walked, distance, consumed calories, and heart rate, were collected by the wearable devices. All participants were taught how to record a dietary diary (taking photos of meals) using a smartphone app. All collected information was uploaded to the health management platform. Only the researchers could access the data on the health management platform.

LINE is a mobile app operated by LINE Corporation. All users can use texts, images, videos, and audio for contact at any time. A LINE group was created to deliver medical knowledge of diet and exercise. The messages were guided by a diet manual for kidney disease (edited by the Department of Dietetics, National Taiwan University Hospital Yunlin Branch) [26].

The intervention involved diet, exercise, and self-management education. The researchers, who had been trained by a dietitian, reminded the intervention group to upload their dietary diary every day and provided suggestions about diet and exercise. In a recent systematic review and meta-analysis, covering 19 studies published from 2005 through 2017, the follow-up duration of studies related to self-management ranged from 3 months (5 studies) to 60 months [27]. This study required daily recommendations of diet and exercise; therefore, we performed the intervention for 90 days. A daily target of 7500 steps was set [28] and used to emphasize the correct concepts about exercise. The LINE app was used to inspire the participants when achieving the target number of steps. Participants in the intervention group also had the opportunity to ask questions about CKD management via the LINE app, and teleconsultations of health information were provided.

Routine care was defined as health education provided by case managers based on the national multidisciplinary pre-ESRD care project and early CKD programs [29]. The case managers offered health guidance during each outpatient visit according to the patient’s renal function and blood test results. At the end of study, we provided every participant with the diet manual for kidney disease, but did not invite the control group to join the LINE group established for the intervention or provide individualized dietary suggestions.

### Statistical Analysis

Statistical analyses were performed using IBM SPSS Statistics for Windows, version 22.0.0 (IBM Corporation), and a two-sided *P* value <.05 was considered statistically significant. The distributional properties of data are expressed as mean (SD) for continuous variables with a normal distribution or as median (IQR) for variables with a skewed distribution. For numerical data, a Student *t* test *was used for comparisons within groups and* between groups; for categorical variables with percentages, the chi-square or Fisher exact test was used. **Analysis of covariance** (ANCOVA), a generalized linear model, was used to analyze the effectiveness of the intervention. The results were analyzed according to the per-protocol principle.

## Results

### Baseline Participant Characteristics

**Figure 3:**
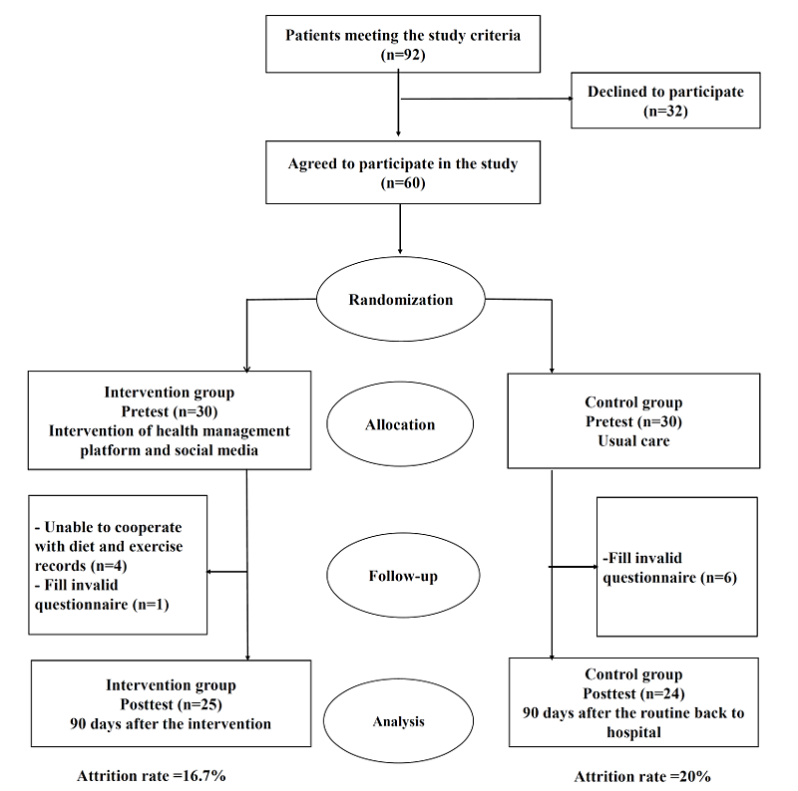
Flow diagram of participant recruitment and randomization.

Figure 3 shows the recruitment process for this randomized controlled trial. A total of 60 participants completed the pretest, with 30 participants in each group. Five patients in the intervention group and six patients in the control group withdrew from the study. A total of 49 participants completed the posttest, including 25 in the intervention group and 24 in the control group. The baseline characteristics of the 11 patients that withdrew from the study did not differ from those of the remaining 49 participants.

The mean age of all participants was 51.22 years (SD 10.98) and 73.5% were men. The baseline characteristics of these participants are presented in Table 1. The patients with stage-2 CKD outnumbered those with stages 3 and 4. There were no differences in comorbidities between groups. The patients in the intervention and control groups had similar levels of serum creatinine. Most of the participants had a bachelor’s degree, although more participants in the intervention group had a university degree compared with the control group.

**Table 1:** Baseline characteristics of the intervention and control groups (N=49).

Characteristic		All (N=49)	Intervention group (n=25)	Control group (n=24)	*P* value
Men, n (%)		36 (74)	17 (68)	19 (79)	.52
Age (years), mean (SD)		51.22 (10.98)	50.60 (11.87)	51.87 (10.20)	.69
Aged ≥65 years, n (%)		4 (8)	3 (12)	1 (4)	.48
**Education level, n (%)**					.02
	Elementary school	2 (4)	0 (0)	2 (8)	
	Junior high school	5 (10)	1 (4)	4 (17)	
	Senior high school	13 (27)	4 (16)	9 (38)	
	College or university	29 (59)	20 (80)	9 (38)	
**Comorbidities, n (%)**					
	Diabetes mellitus	17 (35)	8 (32)	9 (38)	.50
	Hypertension	23 (47)	11 (44)	12 (50)	.39
	Dyslipidemia	32 (65)	16 (64)	16 (67)	.50
**Body composition, mean (SD)**					
	Body weight (kg)	76.27 (14.29)	75.84 (15.52)	76.71 (13.19)	.84
	Body fat percentage	29.39 (5.51)	29.90 (5.93)	28.84 (5.11)	.51
	BMI	27.28 (4.29)	27.07 (4.46)	27.05 (4.19)	.73
	Basal metabolic rate	1638.29 (246.50)	1619.6 (271.51)	1657.75 (221.61)	.59
**Laboratory parameters, mean (SD)**					
	Creatinine (mg/dL)	1.26 (0.40)	1.15 (0.34)	1.33 (0.44)	.12
	eGFR^a^ (mL/min/1.73 m^2^)	66.53 (23.61)	73.03 (25.01)	64.27 (22.72)	.21
**CKD^b^ stage, n (%)**					.46
	1	8 (16)	5 (20)	3 (13)	
	2	22 (45)	11 (44)	11 (46)	
	3a	11 (22)	7 (28)	4 (17)	
	3b	7 (14)	2 (8)	5 (21)	
	4	1 (2)	0 (0)	1 (4)	

^a^eGFR: estimated glomerular filtration rate.

^b^CKD: chronic kidney disease.

### Body Composition and Exercise

As shown in Table 2, there was no difference in baseline body weight in the control and intervention groups (*P*=.84). The baseline BMI and body fat percentage levels were similar between the two groups (*P*=.73 and *P*=.51, respectively). At the end of the study, both groups showed modest weight gains with no differences between groups (*P*=.89). There were no differences in body composition (body fat percentage, basal metabolic rate) between the two groups at the end of the study.

**Table 2:** Comparison of body composition between groups.

Variable	Intervention group (n=25)		Control group (n=24)		ANCOVA^a^			Partial ε^2^		R^2^ (adjusted R^2^)		Power
	Pretest, mean (SD)	Posttest, mean (SD)	Pretest, mean (SD)	Posttest, mean (SD)	*F* value (df=1)	*P* value						
Body weight (kg)	75.84 (15.52)	76.25 (15.52)	76.71 (13.19)	76.80 (13.27)	0.294	.59	0.006		0.982 (0.981)		0.083	
Body fat percentage	29.90 (5.93)	29.44 (6.18)	28.84 (5.11)	29.02 (5.87)	0.970	.33	0.021		0.858 (0.852)		0.161	
BMI (kg/m^2^)	27.07 (4.46)	27.19 (4.45)	27.05 (4.19)	27.73 (3.91)	0.181	.67	0.004		0.918 (0.914)		0.070	
Basal metabolic rate (kJ/m^2^·h)	1619.60 (271.51)	1620.08 (280.31)	1657.75 (221.61)	1648.12 (232.62)	0.325	.57	0.007		0.941 (0.938)		0.086	

^a^ANCOVA: analysis of covariance.

After the 90-day intervention, the steps per day increased in the intervention group (9768.56 in the 1st week and 11,389.12 in the 12th week). Although this difference was not significant (*P*=.10), it indicates a trend of increased physical activity in the intervention group but not in the control group.

### Physiological Indicators After the Intervention

We used ANCOVA to analyze the differences in serum creatinine and eGFR levels between the intervention and control groups after the intervention. As shown in Table 3, serum creatinine levels were lower in the intervention group than in the control group, although the difference was not significant. The eGFR of the intervention group was significantly higher than that of the control group. In other words, the decline in eGFR was significantly slower in the intervention group (–0.56 vs –4.58 mL/min/1.73 m^2^). There were no significant differences between groups in blood glucose levels or uric acid and lipid profiles.

**Table 3:** Comparison of physiological indicators between groups.

Variable	Intervention group (n=25)		Control group (n=24)		ANCOVA^a^		Partial ε^2^	R^2^ (adjusted R^2^)	Power
	Pretest, mean (SD)	Posttest, mean (SD)	Pretest, mean (SD)	Posttest, mean (SD)	*F* value (df=1)	*P* value			
Creatinine (mg/dL)	1.15 (0.34)	1.16 (0.39)	1.33 (0.44)	1.51 (0.81)	1.300	.26	0.027	0.810 (0.801)	0.200
eGFR^b^ (mL/min/1.73 m^2^)	73.03 (25.01)	72.47 (24.28)	64.27 (22.72)	56.69 (22.25)	5.341	.03	0.104	0.921 (0.918)	0.619
Uric acid (mg/dL)	6.28 (1.29)	6.28 (1.14)	6.30 (1.63)	6.70 (1.60)	2.271	.14	0.047	0.575 (0.556)	0.314
T-CHO^c^ (mg/dL)	181.80 (37.57)	179.40 (35.20)	174.42 (43.83)	169.21 (39.54)	0.553	.46	0.012	0.685 (0.672)	0.113
TG^d^ (mg/dL)	170.28 (77.99)	160.20 (85.29)	257.37 (273.39)	272.87 (380.67)	0.225	.64	0.005	0.540 (0.520)	0.075
LDL-C^e^ (mg/dL)	108.24 (29.78)	105.84 (27.86)	95.62 (38.25)	92.29 (31.90)	0.932	.34	0.020	0.840 (0.833)	0.157
GluAC^f^ (mg/dL)	111.04 (39.12)	112.76 (41.91)	118.25 (39.13)	116.25 (28.74)	0.007	.93	0.000	0.396 (0.370)	0.051

^a^ANCOVA: analysis of covariance.

^b^eGFR: estimated glomerular filtration rate.

^c^T-CHO: total cholesterol.

^d^TG: triglyceride.

^e^LDL-C: low-density lipoprotein cholesterol.

^f^GluAC: glucose ante cibum.

### Self-Efficacy and Self-Management

Before the intervention, there was no significant difference in the mean self-efficacy scores between the intervention and control groups; however, after the 90-day intervention, self-efficacy scores were significantly higher in the intervention group (Table 4). Among the self-efficacy subscales, only the infection prevention subscale showed no significant difference between groups. In the other 7 subscales, the intervention group showed significant improvement, especially in blood sugar or blood pressure control, partnership, and lifestyle (Table 4).

The baseline self-management scores also showed no significant difference between the intervention and control groups. However, after the 90-day intervention, self-management scores were significantly higher in the intervention group, and this difference was evident for all 4 subscales (Table 4).

**Table 4:** Comparison of self-efficacy and self-management scores between groups.

Variable		Intervention group (n=25)		Control group (n=24)		ANCOVA^a^		Partial ε^2^	R^2^ (adjusted R^2^)	Power
		Pretest, mean (SD)	Posttest, mean (SD)	Pretest, mean (SD)	Posttest, mean (SD)	*F* value (df=1)	*P* value			
**Self-efficacy scores**										
	Total self-efficacy of CKD^b^	167.36 (30.89)	171.28 (22.92)	154.29(29.04)	142.21 (26.36)	13.728	.001	0.230	0.388 (0.361)	0.952
	Blood sugar or blood pressure control	24.36 (6.24)	26.32 (4.49)	20.58 (6.19)	19.71 (5.49)	15.393	<0.001	0.251	0.534 (0.514)	0.970
	Diet	32.40 (7.37)	31.08 (7.15)	28.96 (7.42)	25.62 (6.14)	6.358	.02	0.121	0.175 (0.140)	0.695
	Exercise	24.08 (6.05)	24.28 (5.25)	20.33 (7.91)	19.29 (6.28)	5.555	.02	0.108	0.308 (0.278)	0.636
	Medical treatment	27.48 (4.11)	28.12 (3.00)	27.29 (4.14)	25.58 (4.22)	5.975	.02	0.115	0.169 (0.133)	0.668
	Lifestyle	17.60 (3.37)	18.04 (2.21)	16.37 (3.63)	14.92 (4.61)	8.865	.005	0.162	0.569 (0.550)	0.830
	Infection prevention	7.12 (3.24)	7.80 (1.91)	7.00 (2.13)	6.96 (2.05)	2.206	.14	0.046	0.119 (0.081)	0.307
	Problem-solving	16.52 (4.71)	17.08 (3.01)	16.50 (3.13)	14.46 (3.69)	7.959	.007	0.147	0.213 (0.179)	0.789
	Partnership	17.80 (4.09)	18.56 (2.20)	17.62 (2.90)	15.67 (3.51)	11.872	.001	0.205	0.220 (0.187)	0.921
**Self-management scores**										
	Total self-management of CKD	52.88 (8.25)	54.16 (6.71)	49.20 (7.04)	47.58 (6.42)	8.929	.004	0.163	0.477 (0.454)	0.833
	Partnership	10.52 (1.85)	10.48 (1.58)	9.79 (1.67)	8.96 (1.71)	7.894	.007	0.146	0.308 (0.278)	0.785
	Compliance	6.80 (1.29)	6.88 (0.93)	6.29 (1.04)	6.17 (0.87)	5.182	.03	0.101	0.298 (0.267)	0.606
	Self-care	28.96 (4.70)	30.16 (3.80)	27.00 (3.97)	27.08 (3.82)	5.170	.03	0.101	0.480 (0.458)	0.605
	Problem-solving	6.60 (1.32)	6.64 (1.29)	6.12 (1.51)	5.37 (1.34)	9.589	.003	0.172	0.355 (0.327)	0.858

^a^ANCOVA: analysis of covariance.

^b^CKD: chronic kidney disease.

### Quality of Life

Before the intervention, the KDQOL-SF scores did not differ significantly between the two groups (*P*=.64); however, after the 90-day intervention, the KDQOL-SF scores were significantly higher in the intervention group (Table 5). The scores for the subscales of physical functioning and medical staff encouragement were significantly higher in the intervention group, with no differences found for the other subscales (Table 5).

**Table 5:** Comparison of self-efficacy and self-management scores between groups.

Variable	Intervention group (n=25)		Control group (n=24)		ANCOVA^a^		Partial ε^2^	R^2^ (adjusted R^2^)	Power
	Pretest, mean (SD)	Posttest, mean (SD)	Pretest, mean (SD)	Posttest, mean (SD)	*F* value (df=1)	*P* value			
All quality of life scales	288.92 (26.19)	293.16 (34.21)	285.04 (31.61)	276.37 (32.21)	5.716	.02	0.111	0.695 (0.681)	0.648
36-item health survey scales	114.68 (13.35)	118.00 (15.64)	108.66 (14.28)	107.63 (15.93)	2.813	.10	0.058	0.574 (0.555)	0.375
General health perceptions	20.12 (3.92)	19.52 (5.12)	18.08 (3.42)	17.75 (3.48)	0.495	.49	0.011	0.175 (0.139)	0.106
Physical functioning	25.88 (3.82)	27.24 (3.18)	24.17 (4.91)	24.04 (4.67)	6.279	.02	0.120	0.549 (0.530)	0.689
Role-physical	7.04 (1.15)	7.40 (1.29)	6.62 (1.69)	6.50 (1.77)	3.807	.06	0.076	0.543 (0.524)	0.480
Role-emotional	5.36 (1.15)	5.60 (0.96)	4.92 (1.31)	5.33 (1.09)	0.031	.86	0.001	0.383 (0.356)	0.053
Social function	8.12 (1.61)	8.44 (1.63)	8.08 (1.66)	8.08 (1.69)	0.651	.42	0.014	0.247 (0.214)	0.124
Pain	9.44 (1.73)	9.40 (1.98)	8.92 (1.95)	8.75 (2.38)	0.234	.63	0.005	0.483 (0.461)	0.076
Emotional well-being	22.00 (2.78)	23.00 (3.38)	22.25 (2.66)	21.75 (3.35)	2.115	.15	0.044	0.127 (0.089)	0.296
Energy/fatigue	16.72 (3.03)	17.40 (3.40)	15.62 (2.96)	15.42 (3.62)	2.182	.15	0.045	0.375 (0.348)	0.304
All kidney disease– targeted scales	174.24 (15.04)	175.16 (19.73)	170.12 (16.81)	168.75 (17.94)	0.600	.44	0.013	0.712 (0.699)	0.118
Burden of kidney disease	14.36 (3.69)	15.36 (3.68)	15.33 (3.96)	15.17 (3.42)	0.912	.35	0.019	0.401(0.375)	0.155
Quality of social interaction	15.48 (2.06)	14.96 (2.65)	13.62 (4.02)	14.58 (2.50)	0.004	.95	0.000	0.083 (0.044)	0.050
Cognitive function	15.28 (1.99)	15.16 (2.28)	14.71 (2.97)	14.79 (2.19)	0.039	.85	0.001	0.263 (0.231)	0.054
Symptom/problems	48.20 (5.31)	48.40 (5.96)	47.67 (5.05)	46.29 (6.35)	1.726	.20	0.036	0.509 (0.488)	0.251
Effects of kidney disease	36.12 (4.30)	35.72 (5.65)	34.83 (5.85)	35.21 (5.76)	0.264	.61	0.006	0.564 (0.545)	0.080
Sleep	19.48 (2.86)	20.28 (3.36)	19.54 (3.20)	18.79 (4.28)	2.632	.11	0.054	0.305 (0.275)	0.355
Social support	6.60 (1.47)	6.24 (1.69)	6.62 (1.34)	6.45 (0.88)	0.342	.56	0.007	0.179 (0.144)	0.088
Work status	3.64 (0.57)	3.60 (0.65)	3.42 (0.65)	3.37 (0.57)	0.393	.53	0.008	0.387 (0.361)	0.094
Patient satisfaction	6.68 (1.41)	6.48 (1.71)	6.01 (1.32)	6.08 (1.14)	0.087	.77	0.002	0.351 (0.323)	0.060
Medical staff encouragement	8.40 (1.19)	8.96 (1.02)	8.17 (1.31)	8.00 (1.38)	8.263	.006	0.152	0.418 (0.393)	0.803

^a^ANCOVA: analysis of covariance.

### Dietary Suggestions and Participant Satisfaction

The investigators provided individualized dietary suggestions online for intervention group participants. The most frequent suggestions included increasing the amount and types of vegetables and fruits, reducing protein intake, and adjusting calories. Most of the participants in the intervention group (19/25, 76%) gave positive feedback about the intervention, and 88% (22/25) reported better changes in dietary and exercise habits. Six of the 25 (24%) participants in the intervention group felt that wearing the smart wristband was inconvenient.

## Discussion

### Principal Findings

This study provides evidence that the use of wearable devices with a health management platform and social media support not only strengthened self-efficacy and self-management but also improved quality of life and slowed the eGFR decline in patients with CKD stages 1-4. These results establish a new self-management model for promoting healthy lifestyle behaviors in patients with CKD. Although Taiwan has implemented preventive programs and patient education for people with CKD since 2006, these programs do not emphasize self-management and are not individualized.

### Self-Management in CKD

Long-term CKD management requires a high level of patient involvement, both in terms of decision-making and in the implementation of care. Patient self-management approaches generally aim to increase self-sufficiency and reduce health care costs.

In a 2019 systematic review and meta-analysis, 19 studies published from 2005 through 2017 were identified, with a total of 2540 CKD patients and a mean follow up of 13.44 months [27]. Compared with usual care, self-management interventions did not show a significant difference in the risk of all-cause mortality or change in eGFR. However, self-management interventions were associated with a lower 24-hour urinary protein excretion level, lower blood pressure level, lower C-reactive protein level, and longer distance on the 6-minute walk when compared with those of controls. These four factors are all known risk factors for cardiovascular disease. Among the 19 studies, the most common intervention type was face-to-face intervention; only 5 studies used a combination of face-to-face and telehealth approaches [30-32]. No investigation in the review used a combination of wearable devices, a health management platform, and social media for an intervention without any face-to-face education, which was adopted as the intervention in our study.

We observed a slower deterioration of renal function following the intervention, which was not consistent with the results of the systematic review. However, several studies within the review showed positive effects of self-management on renal function. In a randomized controlled trial conducted in 2011, Chen et al [32] addressed self-management support in CKD stage 3-5 (mean age of participants 68.2, SD 12.1 years) with an intervention that comprised health information, patient education, weekly telephone-based support, and a support group. After 12 months, the absolute eGFR was significantly higher in the intervention group than that in the control group (29.11, SD 20.61 vs 15.72, SD 10.67 mL/min/1.73 m^2^; *P*=.04), and fewer hospitalization events were noted in participants who received the intervention. In 2018, Wu et al [11] tested an innovative self-management intervention in patients with CKD stages 3b-5 (mean age 70.2, SD 11.6 years). The intervention included a video, a group training manual about self-efficacy and management of CKD, telephone interviews, and small-group interventions (once per week for 1 month). After 3 months, the intervention effectively decreased serum creatinine levels (2.96, SD 2.14 vs 3.04, SD 2.17 mg/dL; *P*=.02) and levels of depression (*P*=.02) in CKD patients [11]. The findings of our study are consistent with those of the two studies summarized above, possibly because of the similarities in the interventions, which used convenient technology and nearly daily contact between researchers and participants.

Our intervention group had better scores in self-efficacy of blood sugar and blood pressure control, which reflects a belief in their ability to take action for their chronic illness. Previous investigations have also shown improvement of health-related quality of life scores when tailored information was provided [33]. This may be related to the sense of feeling supported and empowered [10]. However, the higher self-efficacy scores in this study were not reflected in actual blood glucose levels, possibly because the study period of 90 days was too short.

### Design of the Telehealth Intervention

This interventional study is unique in the combination of a wearable device, a health management platform, and social media in a population with CKD stages 1-4. Few studies have adopted wearable devices, a health management platform, and social media together to quantify motor performance with immediate feedback to empower participants [17]. The use of new technology offers another way to deliver health care. However, it requires technological literacy among the patient population.

Although there is a high prevalence of smartphone use in Taiwan, this study was performed in a rural area, which likely reduces motivation for smartphone use compared to use in an urban area. In addition, the average age of patients with CKD stages 1-4 in the hospital from which the participants were recruited is 69.72 years, whereas the participants in this study were predominantly younger (mean age 51.22 years); they were also open-minded, eager to learn, and aggressive about keeping healthy.

Our intervention type was a multifactorial behavior modification, including exercise and diet. A previous study showed that a 4-month dietary calorie restriction and aerobic exercise intervention resulted in benefits in body weight, fat mass, and markers of oxidative stress and inflammatory response in patients with moderate to severe CKD [34]. Another study found that a dietitian-provided telehealth-delivered regular dietary intervention was well accepted by patients with CKD stages 3-4 [35]; they were more aware of their dietary needs and could prioritize dietary behavior changes. In our study, dietary calorie restriction was not suggested, and body weight change was insignificant. However, we did provide real-time suggestions to the participants according to uploaded images of their current diet, and they could adjust their dietary choices day by day.

The 2015 Taiwan Chronic Kidney Disease Clinical Guidelines [36] encourage physical activity compatible with cardiovascular health and tolerance, aiming for at least 30 minutes of exercise 5 times per week. For CKD patients, there is no limitation on the type of exercise, although strenuous exercise is not recommended. A goal of 10,000 steps per day is commonly believed to be necessary for maintaining good health, but the evidence for this number is lacking. In a recent prospective cohort study of 18,289 women in the United States (aged 72.0, SD 5.7 years), as few as approximately 4400 steps per day was significantly related to lower mortality rates as compared with approximately 2700 steps per day. With more steps per day, mortality rates progressively decreased before leveling off at approximately 7500 steps per day [28]. For this reason, this study set a goal of 7500 steps per day for the participating CKD patients. In our pedometer-guided exercise study, the steps per day tracked by participants were better than our expectations.

During a global pandemic such as COVID-19, telemedicine is a good method of clinical practice to limit travel and exposure, and encourage social distancing while inspiring care from medical staff. For example, chatbots are artificial intelligence (AI) computer programs that simulate text-messaging dialogs with users. The purpose is to help users solve trivial problems in their daily lives, such as ordering food and calling a car. In the near future, AI health advice robots will be widely used to integrate services such as circuit training and diet programs with healthy food, long-term tracking of health indices, personalized AI advice, and social networking. More investigation is needed into the potential effects of AI on CKD management. Regardless of the stage of the disease, maintaining healthy regimes of diet and exercise, and good adherence to medication not only promotes health but also reduces the cost of health care.

### Limitations

Participants in this study were recruited from one teaching hospital in central Taiwan. In addition, the sample was relatively small because of the limited number of wearable devices available. At baseline, we found a significant difference in educational level between the intervention and control groups. Education might thus be a major confounder for the results obtained. In an 11-year follow-up study of the Dutch general population, a low educational level was associated with an elevated risk of CKD [37]. This association is suggested to be driven by higher rates of diabetes and modifiable risk factors such as abdominal obesity, smoking, low potassium intake, and hypertension in those with lower education. In a US study (with a median follow-up time of approximately 23 years), socioeconomic status (annual household income, educational attainment, or neighborhood deprivation) was associated not only with ESRD risk but also with eGFR decline, although the association with CKD appeared to be weaker [38]. This suggests that observing the effects of educational level and socioeconomic status takes time. In this study, given that the follow-up period was only 3 months, the effect of the difference in educational level is considered to be limited.

Although self-efficacy, self-management, and quality of life outcomes improved after the 90-day intervention, the long-term effect of the intervention should also be further evaluated. The effectiveness of an intervention on body composition and renal function cannot be well evaluated over a short period of time. A follow-up period of at least 1 year would allow for better evaluation of the long-term effectiveness of the intervention.

Finally, our study and intervention design made it impossible to distinguish between the effects of exercise, dietary intervention, or emotional support via social media.

### Conclusion

A self-management intervention that combines wearable devices, a health management platform, and social media could strengthen self-efficacy and self-management, and lead to improvements in quality of life for people with CKD stages 1-4. The effects of this nonpharmacologic intervention were also reflected in a slower decline in eGFR. These results outline a self-management model that can promote healthy lifestyle behaviors in patients with CKD.

## Abbreviations

AI: artificial intelligence
ANCOVA: analysis of covariance
CKD: chronic kidney disease
eGFR: estimated glomerular filtration rate
ESRD: end-stage renal disease
KDQOL-SF: Short Form Kidney Disease Quality of Life
mHealth: mobile health
